# Amyotrophic lateral sclerosis (ALS) linked mutation in Ubiquilin 2 affects stress granule assembly via TIA‐1

**DOI:** 10.1111/cns.13757

**Published:** 2021-11-08

**Authors:** Guangnan Peng, Ao Gu, Hongyan Niu, Linlin Chen, Yan Chen, Miaojin Zhou, Yiti Zhang, Jie Liu, Licong Cai, Desheng Liang, Xionghao Liu, Mujun Liu

**Affiliations:** ^1^ Center for Medical Genetics & Hunan Key Laboratory of Medical Genetics School of Life Sciences Central South University Hunan China; ^2^ Center for Regenerative Medicine The First People’s Hospital of Yunnan Province Kunming China; ^3^ School of Life Sciences Central South University Hunan China; ^4^ Hunan Key Laboratory of Basic and Applied Hematology Central South University Hunan China; ^5^ Hunan Key Laboratory of Animal Models for Human Diseases Central South University Hunan China; ^6^ Department of Cell Biology School of Life Sciences Central South University Hunan China

**Keywords:** amyotrophic lateral sclerosis, protein aggregation, stress granules, TIA‐1, UBQLN2

## Abstract

**Aims:**

The ubiquilin‐like protein ubiquilin 2 (UBQLN2) is associated with amyotrophic lateral sclerosis and frontotemporal degeneration (ALS/FTD). The biological function of UBQLN2 has previously been shown to be related to stress granules (SGs). In this study, we aimed to clarify the regulatory relationship between UBQLN2 and SGs.

**Methods:**

In this study, we transfected UBQLN2‐WT or UBQLN2‐P497H plasmids into cell lines (HEK293T, HeLa), and observed the process of SG dynamics by immunofluorescence. Meanwhile, immunoblot analyses the protein changes of stress granules related components.

**Results:**

We observed that ubiquilin 2 colocalizes with the SG component proteins G3BP1, TIA‐1, ATXN2, and PABPC1. In cells expressing WT UBQLN2 or P497H mutants, in the early stages of SG formation under oxidative stress, the percentage of cells with SGs and the number of SGs per cell decreased to varying degrees. Between WT and mutant, there was no significant difference in eIF2α activity after stress treatment. Interestingly, the UBQLN2 P497H mutant downregulates the level of TIA‐1. In addition, the overexpression of the UBQLN2 P497H mutant inhibited the phosphorylation of 4E‐BP1 and affected the nucleoplasmic distribution of TDP‐43.

**Conclusions:**

Ubiquilin 2 colocalizes with the SG component proteins G3BP1, TIA‐1, ATXN2, and PABPC1. It participates in regulating SG dynamics. And UBQLN2 mutation affects the assembly of stress granules by regulating TIA‐1. In addition, the overexpression of the UBQLN2 P497H mutant inhibited the phosphorylation of 4E‐BP1 and affected the nuclear and cytoplasmic distribution of TDP‐43. These provide new insights into the role of UBQLN2 in oxidative stress and the pathogenesis of ALS.

## Graphical abstract

Stresses activate eIF2‐Á kinases that phosphorylate eIF2‐Á, deplete the ternary complex, and promote the assembly of a noncanonical Pre‐initation complex£¨PIC£©; these interfaces recruit RNA‐BPs such as TIA‐1, increasing the local concentration of these proteins to promote LLPS and assembly of SG seeds. However, UBQLN2 P497H could repress the level of TIA‐1, thereby inhibiting stress granule assembly.

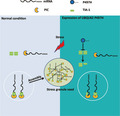

## 1 INTRODUCTION

Amyotrophic lateral sclerosis (ALS), also known as Lou Gehrig's disease, is an adult‐onset neurodegenerative disease caused by loss of motor neurons in the brain and spinal cord.^1^, ^2^ ALS is primarily characterized by progressive degeneration and atrophy of voluntary skeletal muscles, ultimately resulting in paralysis and death from respiratory failure.^3^ The incidence of ALS is approximately 1–2.6 cases per 100,000 persons annually.^4^ Only approximately 5%–10% of ALS cases are familial (fALS), with an autosomal dominant pattern of inheritance and at least 40 genes and loci that exert a significant effect known to contribute to familial pathology.^5^, ^6^ In 2011, missense mutations in the *UBQLN2* gene were identified in large ALS and ALS/FTD families.^7^

Ubiquilin 2 is a member of the ubiquilin family (ubiquilins), which regulates the degradation of ubiquilinated proteins.^8^ More recently, the P497H mutation in *UBQLN2* was found to be associated with dysfunction of the ubiquilin proteasome system, neuro inflammation,^9^ and the formation of stress granules (SGs).^10^ Functional analysis showed that P497H mutation could impair protein degradation and cause abnormal accumulation of proteasomal subunits, such as PSMC2.^11^ In addition, the P497H mutation can also affect autophagy pathway degradation by regulating liposomal acidity.^12^, ^13^

Stress granules are cytoplasmic RNA granules that form when untranslated mRNPs (ribonucleoprotein particles) accumulate in cells subjected to biotic stress or environmental stress.^14^ Abnormal formation of stress granules has been linked to the pathogenesis of neurodegenerative disorders. Mutations in a series of RNA‐binding protein (RBP) genes (*TDP*‐*43*, *FUS*, *hnRNPA1*, *ATXN2*, *TIA*‐*1*) have been confirmed to cause ALS and/or FTD.^15^, ^16^, ^17^ Most of the disease‐causing mutations map to the low complexity domain regions of SG‐related RBPs and disrupt their biophysical properties, leading to increased LLPS (liquid‐liquid phase separation) into SGs and aggregate formation.^18^ The LLPS of UBQLN2 under certain environmental conditions is similar to that of RNP particles. A group recently suggested that UBQLN2 modulates the state of the components to be recruited to SGs. It has recently been reported that overexpression of UBQLN2 mutants (P497H and P506T) in HeLa cells directly decreased the interaction between UBQLN2 and FUS, which resulted in loss of the ability of UBQLN2 to regulate FUS‐RNA complexes and SG formation.^19^ However, the effect of UBQLN2 on the dynamic process and the disturbance of stress granules is still unknown.

Here, we found that UBQLN2 colocalized with SG component proteins, such as G3BP1, TIA‐1, ATXN2, and PABPC1. In cells expressing WT UBQLN2 or the P497H mutant, both the percentage of cells with SGs and the number of SGs per cell were significantly decreased under oxidative stress. P497H mutation led to rapid disassembly of SGs. In addition, overexpression of P497H mutant inhibited the phosphorylation of 4E‐BP1. Importantly, WT UBQLN2 and the P497H mutant downregulated the level of the SG component protein TIA‐1 to different degrees, which provides new insight into the role of UBQLN2 in the negative regulation of SG formation.

## 2 MATERIALS AND METHODS

### 2.1 Cell culture

We maintained HEK293 (national collection of authenticated cell cultures, GNHu 18) 、HEK293T (national collection of authenticated cell cultures, GNHu 17)and HeLa (national collection of authenticated cell cultures, TCHu187) cells in Dulbecco's modified Eagle's medium (DMEM, Thermo Fisher) containing 10% fetal bovine serum, 1% penicillin, and streptomycin solution at 37°C in 5% CO_2_. Cells were plated 24 h before transfection. Following the manufacturer's protocols, HeLa or HEK293 cells were transiently transfected with either green fluorescent protein (GFP)‐UBQLN2‐WT or (GFP)‐UBQLN2‐P497H using Lipofectamine 2000 reagent (Invitrogen). Transfections were carried out using OPTI‐MEM (Gibco) according to the manufacturer's protocol. Transfected cells were incubated for 48 h to allow transient protein expression. After 48 h, the cells were treated with 0.5 mM sodium arsenite (SA, Sigma‐Aldrich) for 0.5 h or incubated at 45°C for 30 min for the stress experiment.

### 2.2 Immunofluorescence labeling

For immunofluorescence, cells were washed three times with DPBS and then fixed using 4% paraformaldehyde (PFA) for 15 min, treated for 15 min with 0.01% PBST, later, cells were washed with DPBS for 5 min and blocked for 1 h using 5% bovine serum albumin (BSA) and 0.1% Triton X‐100 in DPBS and incubated with primary antibody overnight at 4°C. Mouse polyclonal antibody to G3BP1 (1:200, Abcam) or rabbit monoclonal antibody to TIA‐1 (1:200, Santa Cruz Biotechnology), rabbit polyclonal antibody to ATXN2 (1:200) and rabbit polyclonal antibody to PABPC1 were diluted 5% bovine serum albumin (BSA) and 0.1% Triton X‐100 in DPBS. Cells were washed three times with DPBS and incubated with Alexa Fluor (488/Cy3) fluorescently labeled secondary antibodies (1:250) and DAPI in blocking buffer for 1 h at room temperature. Images were acquired with a LAS X SP‐5 confocal microscope (Leica) with a 63X silicon objective. To examine SG formation, images were obtained from different areas of the coverslips, and the total numbers of transfected cells and cells showing cytoplasmic aggregates with G3BP1 (Abcam, ab56574) were determined.

### 2.3 Western blot analysis

Cells were lysed in RIPA buffer containing 50mM Tris (pH 7.4), 150 mM NaCl, 1% Triton X‐100, 1% sodium deoxycholate, 0.1% SDS, sodium orthovanadate, sodium fluoride, EDTA, leupeptin, and protease inhibitor. According to the manufacturer's protocol, protein concentrations were determined using a Pierce BCA protein assay kit (Thermo scientific). Proteins were denatured at 99°C for 10 min with Laemmli sample buffer containing β‐mercaptoethanol. Western blot analysis was performed as described. Western blots were quantified with the Image plug in Gel Analyzer.

### 2.4 Data analysis

GraphPad Prism software (San Diego, CA, USA) was used. The data are presented as the mean ± standard error (SE) from more than three or four independent tests. The D'Agostino & Pearson test or Shapiro‐Wilk test was used to test for normality. If the data passed the normality test, we use the Student's *t*‐test or the One‐way ANOVA to determine the statistical significance of the data. If not, we use the rank sum test. Statistically significant *p* values were lower than 0.05 and are marked with an asterisk.

## 3 RESULTS

### 3.1 UBQLN2 is Localized to SGs

**FIGURE 1:**
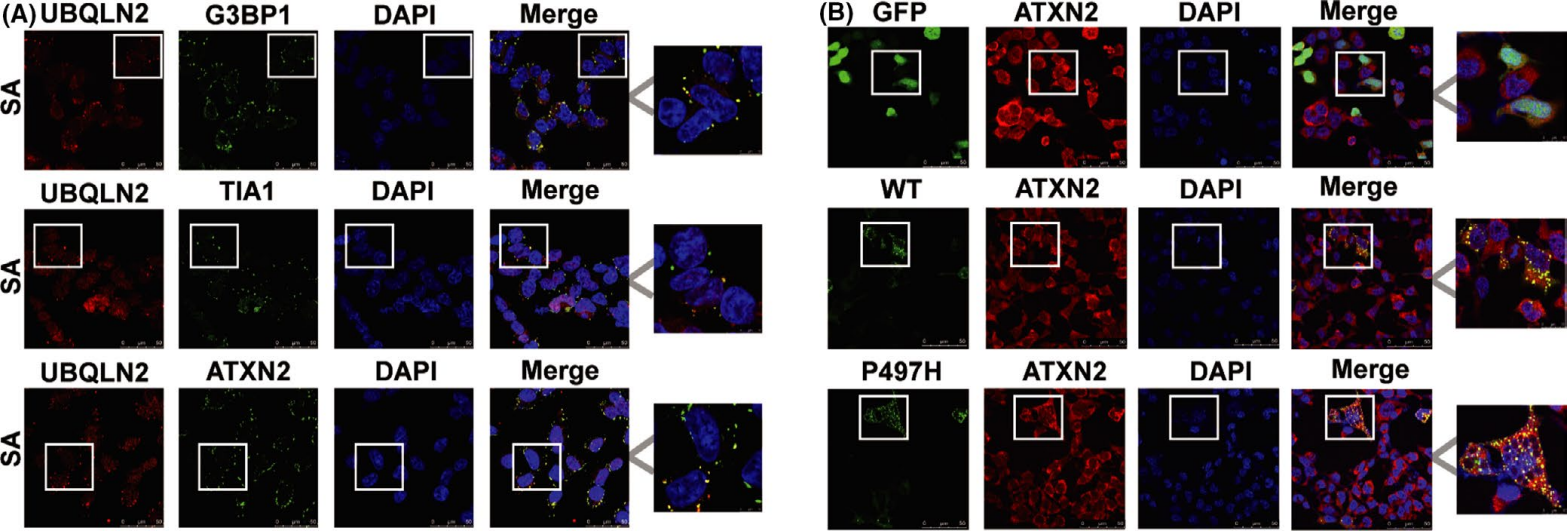
Colocalization of UBQLN2 and SG component proteins. (A) Immunofluorescence showed that endogenous UBQLN2 was colocalized with the SG components G3BP1, TIA‐1, and ATXN2. (B) HEK293T cells were transfected with GFP, UBQLN2‐WT, or UBQLN2‐P497H plasmid, and the colocalization of UBQLN2 with ATXN2 was detected by immunofluorescence. Regions within the boxes are magnified in the figure. Scale, 50 μm

There are many components of SGs, including G3BP1, TIA‐1, TIA‐R, ATXN2, and PABPC1.^20^, ^21^, ^22^ To investigate whether UBQLN2 associates with SG components, we performed an immunostaining experiment. Under sodium arsenite (SA) treatment, endogenous UBQLN2 was shown to colocalize with the SG components G3BP1, TIA‐1, and ATXN2 in HEK293 cells (Figure 1A). To further confirm the relationship between exogenous UBQLN2 and SGs, HEK293T cells were transfected with UBQLN2‐WT or UBQLN2‐P497H plasmid. Immunofluorescence results showed that overexpressed UBQLN2‐WT and UBQLN2‐P497H also colocalized with the SG component ATXN2 (Figure 1B). As some SG components depend on cell lines and stress conditions, we then determined whether UBQLN2 localizes to SGs in HeLa cells. UBQLN2 was shown to colocalize with the SG components G3BP1, TIA‐1, ATXN2, and PABPC1 in HeLa cells under conditions of both SA treatment and heat shock (Figure S1). Based on these observations, UBQLN2 appears to generally localize to SGs.

### 3.2 UBQLN2 affects SG dynamics

**FIGURE 2:**
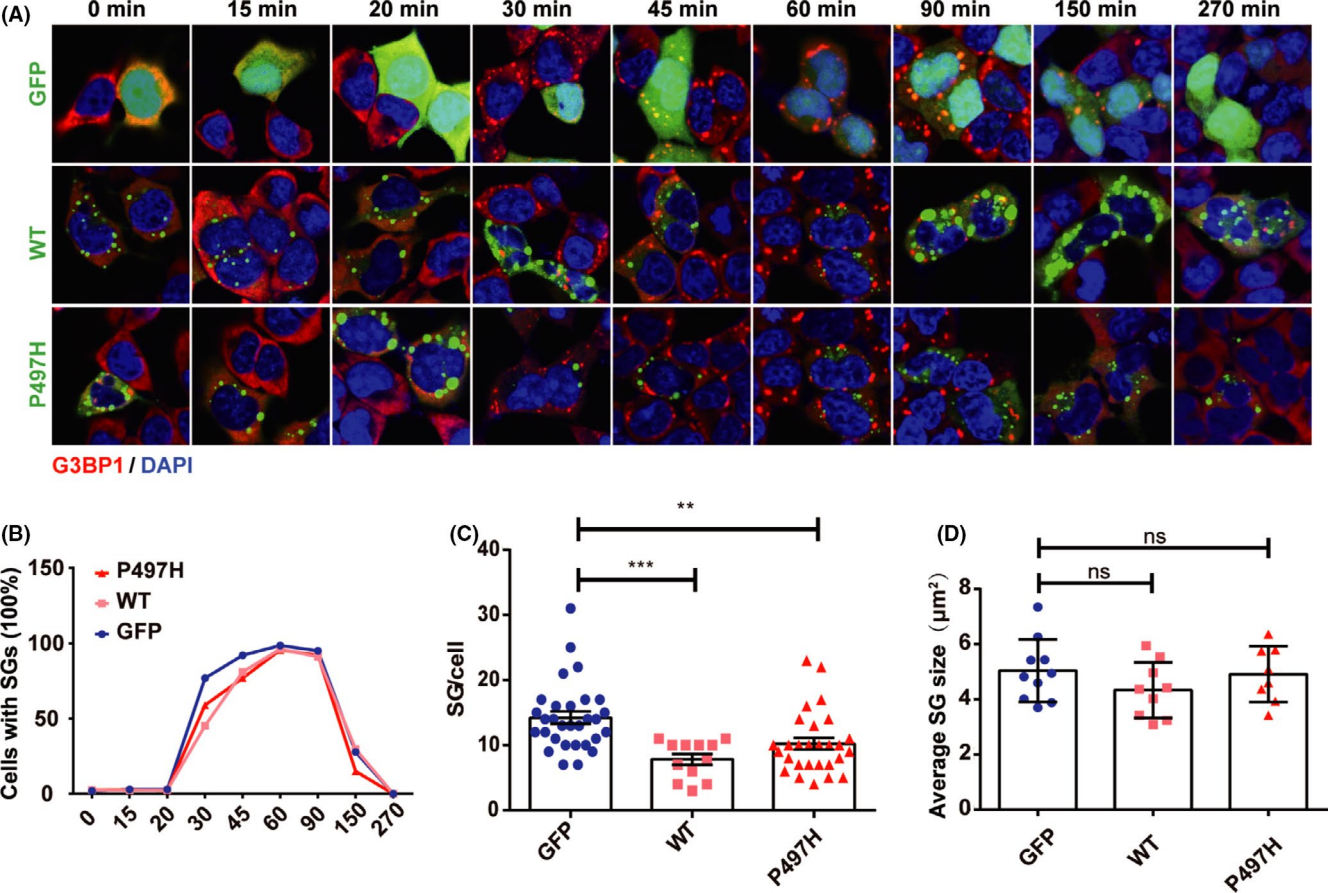
Dynamics of SGs regulated by UBQLN2. (A) HEK293T cells were transfected with GFP, WT, or P49H plasmid. Cells were treated for 15 min, 20 min, or 30 min and recovered for 270 min (A). G3BP1 is a marker protein of SGs. Scale, 10 μm. (B) The positive rate of SGs in the process of SG formation and depolymerization. We randomly selected 100 cells. (C) Significant differences in the number of SGs per cell were observed after 30 min of SA treatment. We counted the number of SG particles in 25 randomly selected transferred cells. (D) We randomly selected ten cells to calculate statistics on the average size of SGs. The cells were treated with SA for 30 min. One‐way ANOVA was used to test the significance of Tukey's test results. ***p < 0.001; **p < 0.01; ns, not significant

Given that UBQLN2 associates with SGs, we questioned whether UBQLN2‐WT and the UBQLN2 mutant impact the process of SG dynamics. To measure the SG assembly kinetics, HEK293T cells were transfected with UBQLN2‐WT‐GFP or UBQLN2‐P497H‐GFP and treated with SA for 15 min, 20 min, and 30 min, followed by fixation and immunostaining for GFP and G3BP1 (Figure 2A). After 30 min of SA treatment, a significant decrease in the percentage of cells that formed SGs was observed in cells expressing either UBQLN2‐WT‐GFP (45.2%) or UBQLN2‐P497H‐GFP (58.9%) compared to control cells (76.8%) (Figure 2B). To measure SG disassembly kinetics, cells were treated with SA for 30 min, leading to robust SG formation in transfected and untransfected cells, followed by SA washout and a recovery period of 45 min, 60 min, 90 min, 150 min, and 270 min (Figure 2A). We observed that the disassembly of SGs in UBQLN2‐P497H cells was accelerated such that 15.0% of cells had SGs at 150 min, compared to control (27.8%) and UBQLN2‐WT (29.7%) cells (Figure 2B). Furthermore, we observed a significant reduction in the number of SGs per cell in both UBQLN2‐WT and UBQLN2‐P497H cells compared to the control cells after 30 min of SA treatment. The number of SGs per cell in UBQLN2‐WT cells was less than that in UBQLN2‐P497H cells (Figure 2C). We observed no significant impact of UBQLN2‐WT or UBQLN2‐P497H on the average size of SGs (Figure 2D). We then expanded our analysis to HeLa cells treated with SA or heat shock (HS) to exclude the possibility that the observed response was cell line‐specific or stress‐specific. In HeLa cells treated with SA or HS, overexpression of WT and P497H reduced the number of SGs per cell after 30 min of treatment (Figure S2A–E). There was no significant difference in the average size of SGs (Figure S2F, G). These experiments show that UBQLN2 overexpression influences SG dynamics and that UBQLN2 P497H had a more significant influence on SG disassembly than UBQLN2‐WT.

### 3.3 UBQLN2 P497H influences the level of TIA‐1

**FIGURE 3:**
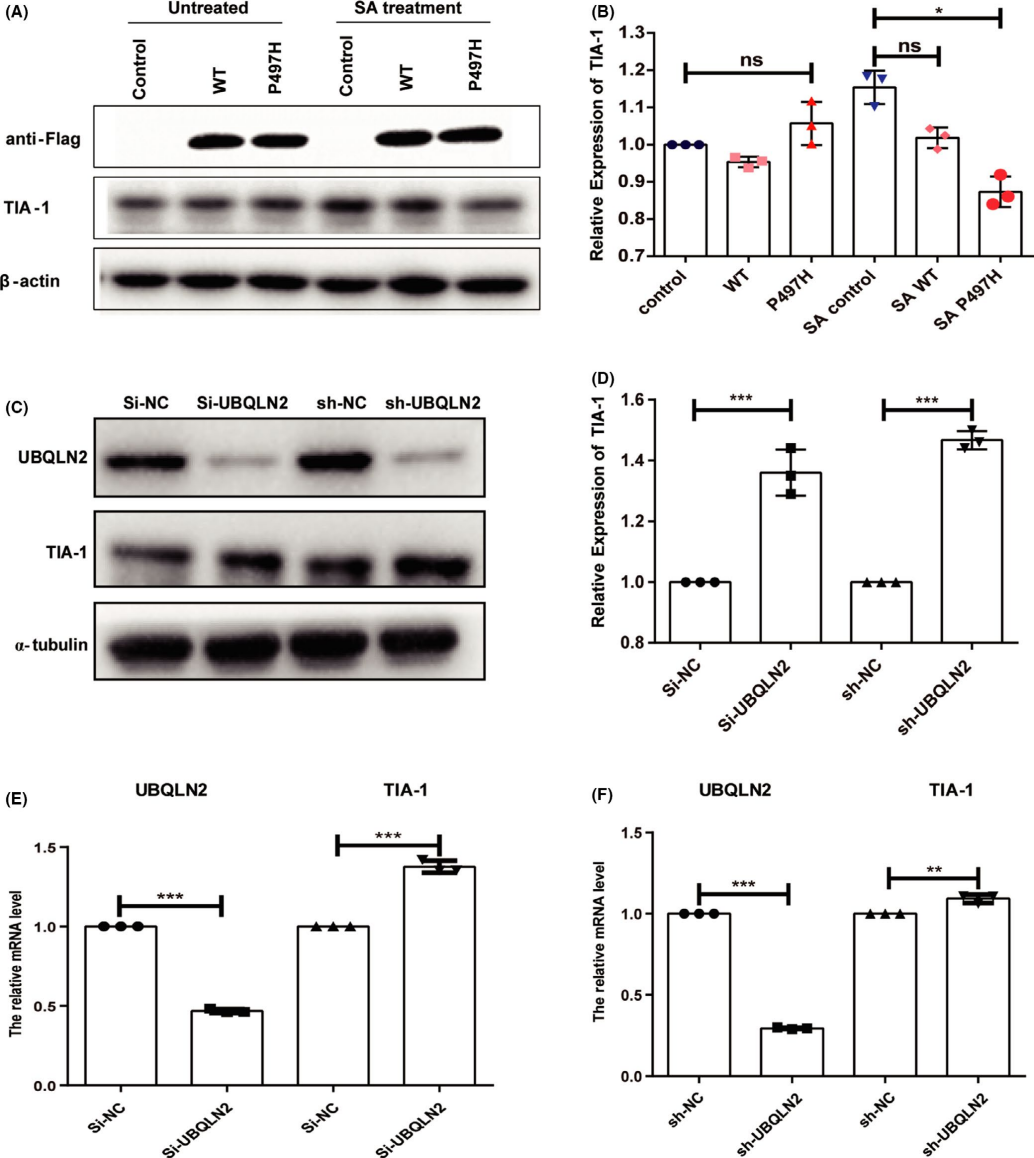
UBQLN2 level influences the expression of TIA‐1. (A, B) After SA treatment for 30 min, the expression level of TIA‐1 in the control (N‐flag), WT (UBQLN2‐flag), and P497H (UBQLN2‐P497H‐flag) mutant groups was detected by immunoblotting. (C) The mRNA relative expression level of UBQLN2 after knockdown UBQLN2. (D)Transfection after 48 h, the expression level of TIA‐1 in the control (si‐NC/sh‐NC) or UBQLN2 shRNA (sh‐UBQLN2), UBQLN2 siRNA(si‐UBQLN2) was detected by immunoblotting. Quantification of the mRNA (F) and protein levels (E) of TIA‐1 after knockdown of UBQLN2. One‐way ANOVA was used to test the significance of Tukey's test results. *p < 0.05; ***p < 0.001; ns, not significant

Since UBQLN2‐WT and UBQLN2‐P497H negatively regulate SG formation, we sought to determine whether UBQLN2‐WT and UBQLN2‐P497H affect the expression of SG‐nucleating proteins. TIA‐1 is a component protein of SG, which can promote the assembly of stress granules.^23^ HeLa cells were transfected with UBQLN2‐WT‐GFP or UBQLN2‐P497H‐GFP plasmid. Immunoblot analysis of SG‐nucleating proteins revealed that the levels of TIA‐1 remained unchanged compared with the level in control cells. Intriguingly, we noted that TIA‐1 was downregulated 19% in UBQLN2‐P497H‐transfected cells after SA treatment (Figure 3A, B). We also used small interference RNA (siRNA) or small hairpin RNA (shRNA) vector to knockdown UBQLN2 (Figure 3C). Immunoblot results showed that the level of TIA‐1 was upregulated (Figure 3C, D), and at the same time, the mRNA level of TIA‐1 was also upregulated (Figure 3E, F). Meanwhile, the level of another SG‐related protein, G3BP1, was not influenced in UBQLN2‐WT and UBQLN2‐P497H cells in the presence and absence of SA treatment (Figure S3A, B). After that, we used shRNA to knockdown TIA‐1 in HEK293T cells and found that the SGs formation rates and the average number of SGs per cell decreased as expected. Knocking down TIA‐1 in the cells with overexpression of UBQLN2 WT or P497H, the SGs formation rates and the average number of SGs were reduced compared to those with UBQLN2 overexpression only (Figure S4). Thus, these results suggest that UBQLN2‐P497H affects the SG assembly by regulating the level of the critical SG protein TIA‐1 upon treatment with SA.

### 3.4 UBQLN2 does not affect eIF2α phosphorylation but regulates 4E‐BP1 phosphorylation

**FIGURE 4:**
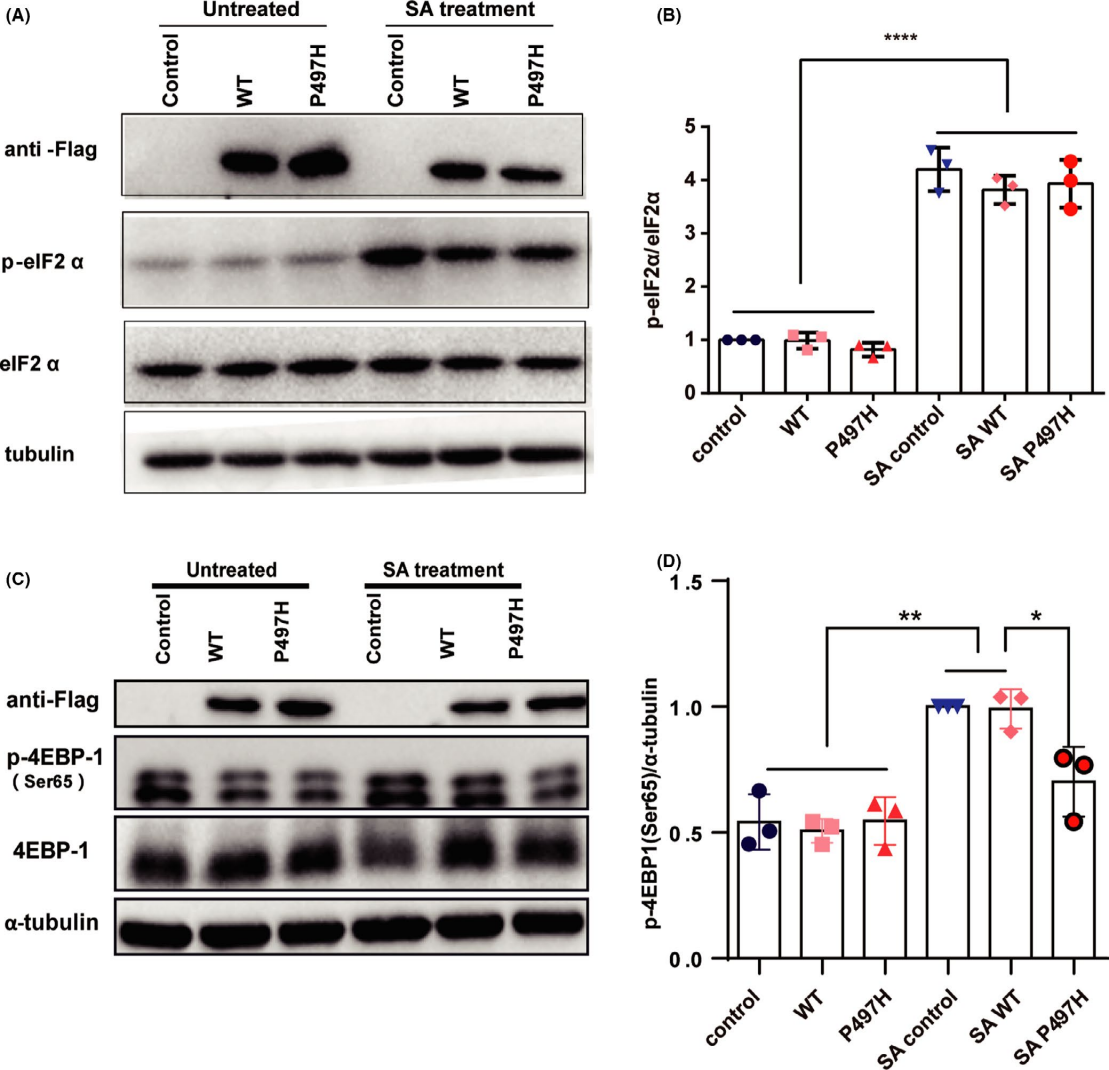
Regulation of the eIF2α/4E‐BP1 signaling pathway by UBQLN2. (A, B) The expression levels of p‐eIF2α and eIF2α in the control (N‐flag), WT (UBQLN2‐flag), and P497H (UBQLN2‐P497H‐flag) groups were detected via western blotting. A statistical graph of p‐eIF2α expression relative to eIF2α expression is shown. (C, D) Expression of phosphorylated 4E‐BP1 was detected via western blotting after transfection with control (N‐flag), WT (UBQLN2‐flag) or P497H (UBQLN2‐P497H‐flag) plasmid. One‐way ANOVA was used to test the significance of Tukey's test results. *p < 0.05; **p < 0.01; ***p < 0.001; ns, not significant

Phosphorylation at serine 51 of eIF2α is involved in the early initiation step in SGs assembly. Phospho‐eIF2α (phosphorylation of eIF2α) inhibits protein synthesis, and a large amount of mRNA and protein are recruited into the stress granules.^24^ To explore whether UBQLN2 can regulate the initial stage of SG formation via the eIF2α signaling pathway, we detected the levels of phospho‐eIF2α and eIF2α in UBQLN2‐WT and UBQLN2 P497H‐transfected HeLa cells in the presence and absence of SA treatment. Following SA exposure, our results showed that phospho‐eIF2α was significantly increased, indicating the stress response of cells after SA treatment. However, there was no difference between the control and UBQLN2‐transfected cells with or without SA exposure (Figure 4A, B), suggesting that the impact of UBQLN2 on SG formation may be independent of phospho‐eIF2α signaling pathways. eIF4E‐binding protein 1 (4E‐BP1) can interact with eIF4E and block translation initiation. Hypophosphorylated 4E‐BP1 inhibits the formation of SGs, while phosphorylated 4E‐BP1 promoting it.^25^ Under normal growth conditions, 4E‐BP1 is phosphorylated by mTOR.^12^ To investigate whether UBQLN2‐WT and UBQLN2‐P497H can affect the phosphorylation of 4E‐BP1, we performed immunoblotting and found that the level of phosphorylated 4E‐BP1 (Ser65) was significantly increased after SA treatment in all groups including control group. Intriguingly, phosphorylation of 4E‐BP1 in UBQLN2‐P497H cells was significantly reduced than UBQLN2‐WT and control group after SA treatment. (Figure 4C, D). In addition,we found that 4E‐BP1 accumulated in cytoplasmic puncta positive for SG marker G3BP1, and nearly all SGs contained 4E‐BP1 (Figure S5). This phenomenon suggests that overexpression of UBQLN2‐P497H may modulate SG formation by regulating 4E‐BP1 phosphorylation.

### 3.5 UBQLN2 impacts the nucleoplasmic distribution of TDP‐43

**FIGURE 5:**
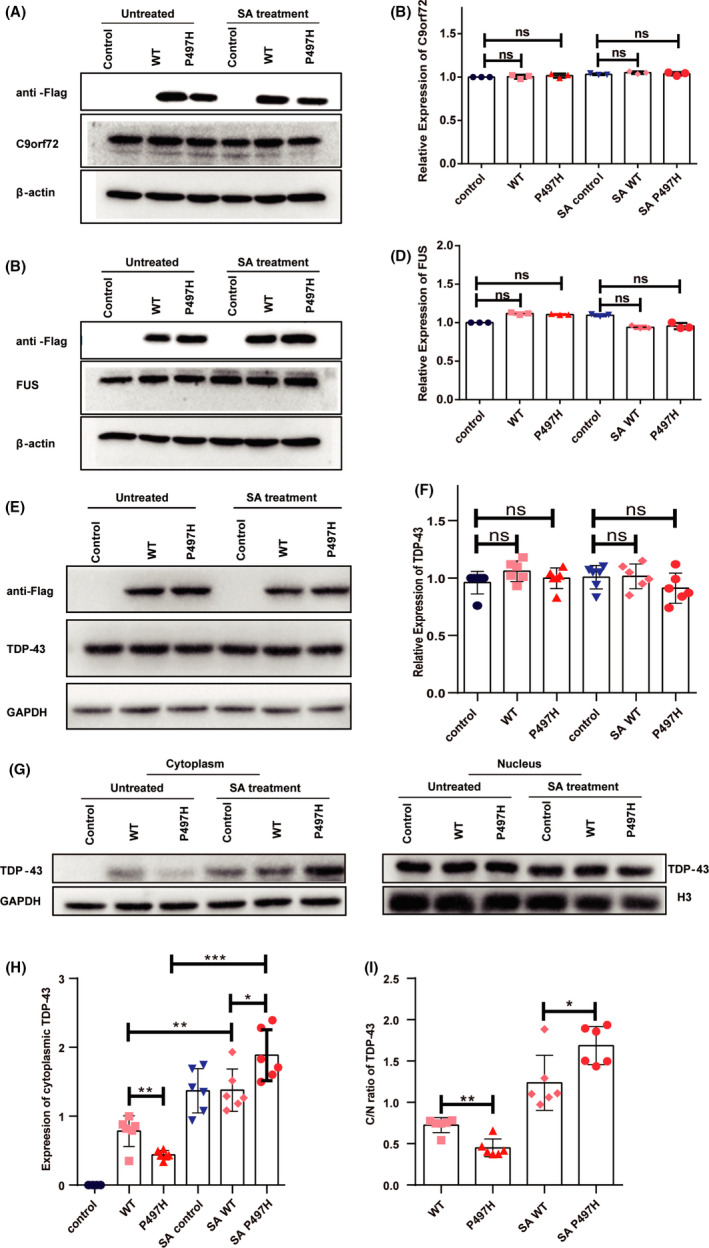
Detection of SG‐related proteins. (A‐F) The expression levels of C9orf72 (A, B), FUS (C, D) and TDP‐43 (E, F) were detected via western blotting after SA treatment. (G, H) The nucleoplasmic distribution of TDP‐43 was detected through a nucleo‐cytoplasmic separation experiment, with GAPDH as the cytoplasmic reference and H3 as the intra‐cellular reference. (I) Relative quantitative analysis of the cytoplasmic/nuclear TDP‐43 ratio (C/N ratio) in UBQLN2‐WT and UBQLN2‐P497H cells in the presence and absence of SA treatment. One‐way ANOVA was used to test the significance of Tukey's test results. *p < 0.05; ns, not significant

*TDP*‐*43*, *FUS*, and *C9ORF72* are pathogenic genes in ALS and significantly affect the dynamic process of SG formation.^14^ Moreover, UBQLN2 has an interactive relationship with these genes. Therefore, to investigate whether overexpression of UBQLN2 affects the expression of these genes, cells were transfected with UBQLN2‐WT or UBQLN2‐P497H plasmid. Protein lysates of UBQLN2‐transfected cells were immunoblotted for TDP‐43, FUS, and C9ORF72. Our results showed that the levels of C9orf72 remained unchanged in UBQLN2‐WT and UBQLN2‐P497H cells in the presence and absence of SA treatment (Figure 5A, B). However, a trend toward downregulation of TDP‐43 and FUS was observed in UBQLN2‐WT cells and UBQLN2‐P497H cells but did not reach statistical significance (Figure 5C‐F). TDP‐43 is a nuclear protein, and cytoplasmic aggregation of TDP‐43 is a pathological marker of ALS.^24^ We analyzed the subcellular distribution of TDP‐43 in UBQLN2‐transfected cells with or without SA exposure (Figure 5G). We found that the level of cytoplasmic TDP‐43 increased in UBQLN2‐WT and UBQLN2‐P497H cells compared with control cells before SA treatment (Figure 5H). Importantly, after SA treatment, the level of cytoplasmic TDP‐43 was increased in all groups, and the degree of relocalization of TDP‐43 into the cytoplasm in the UBQLN2 P497H group was much greater than that in the control and UBQLN2‐WT groups (Figure 5H, I). Taken together, our data indicate that UBQLN2‐P497H affects the nucleoplasmic distribution of TDP‐43.

## 4 DISCUSSION

Here, we investigated the influence of overexpressed wild‐type UBQLN2 and the P497H mutant on SG dynamics and found that they both inhibited the formation of SGs. It should be noted that the overexpression of UBQLN2 in animal models or cells reproduces protein aggregation. In this study, we found that UBQLN2 aggregation increased with culture time, and the aggregation reproduced by the P497H mutant (^>2μm2^) was much greater than that reproduced by the wild‐type strain (Figure S6A, B). Overexpression of UBQLN2 causes neuronal death in rodent models.^9^ Therefore, the impact of UBQLN2 on the formation of SGs may be related to the aggregates induced by overexpression of wild‐type UBQLN2 and the P497H mutant. We also observed that the disassembly of SGs in UBQLN2‐P497H was accelerated compared to that in the control at 150 min (Figure 2B). Other groups have shown that the P497H mutation impacts the protein solubility and ubiquitylation and that the mutated protein can bind more ubiquilin proteins.^26^ However, the specific binding between ubiquilin and UBQNL2 can pull UBQLN2 out of SGs,^10^ which may direct ubiquilination protein to the proteasome for degradation. Therefore, we speculate that the P497H mutant promotes the disassembly of SGs because of the ability of UBQLN2 to combine with more ubiquilin proteins.

**FIGURE 6:**
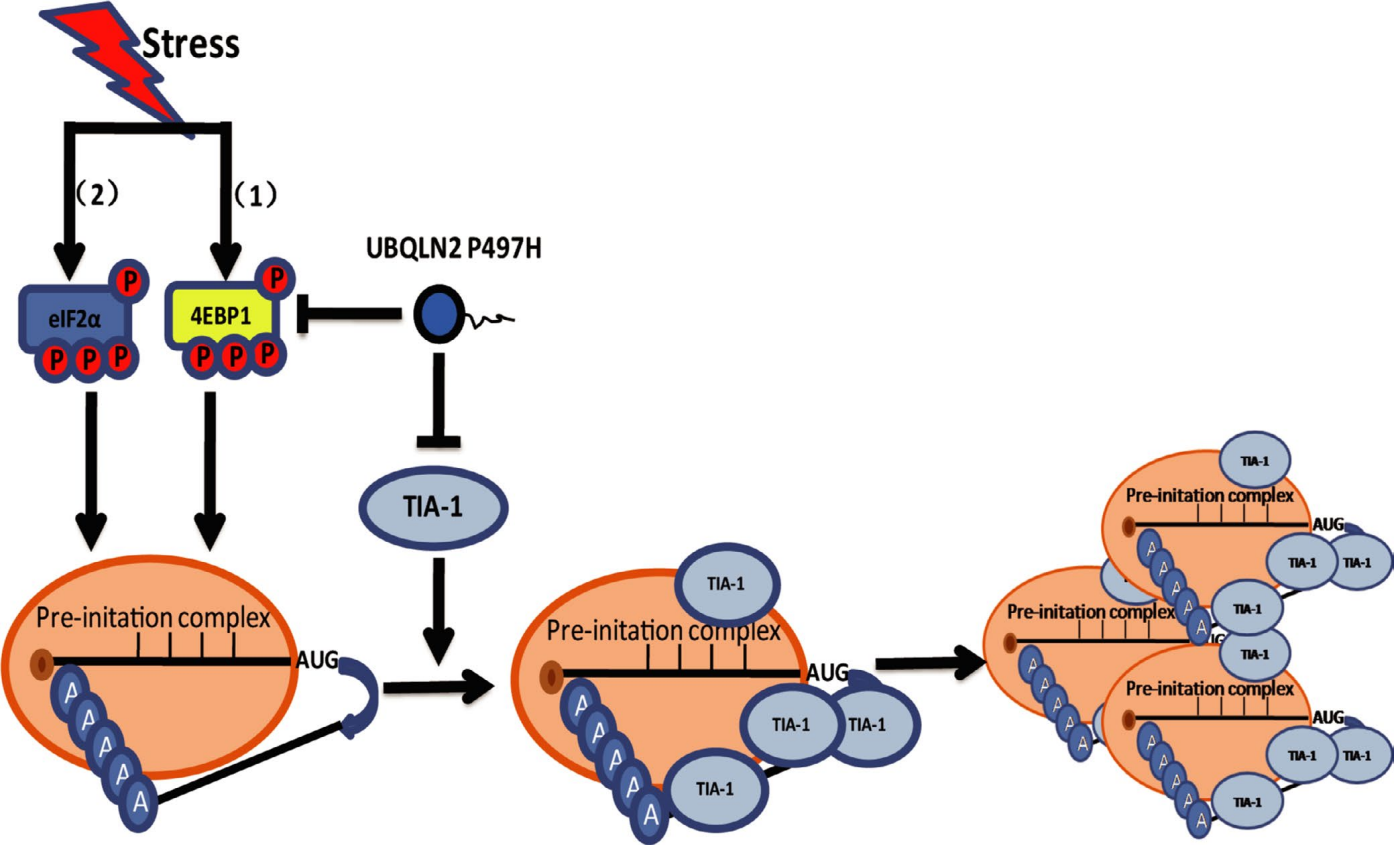
Model of TIA‐1 affects stress granule assembly. (1) Sodium selenite activates mTOR, leading to increased phosphorylation of 4E‐BP1 and blocking translation initiation. (2) Stresses activate eIF2α kinases that phosphorylate eIF2α, deplete the ternary complex, and promote the assembly of a noncanonical Pre‐initation complex (PIC); these noncanonical PICs differ from canonical PICs in composition and exposure of the 40S subunit interface and mRNA. These interfaces recruit RNA‐BPs such as TIA‐1, increasing the local concentration of these proteins to promote LLPS and assembly of SG seeds. However, UBQLN2‐P497H could repress the level of TIA‐1, thereby inhibiting stress granule assembly

TIA‐1, a cytotoxic T lymphocyte granule‐associated RNA‐binding protein, is a critical component of stress granules, which can promote the assembly of stress granules.^23^ Similar reports revealed that knockout of *TIA*‐*1* can make cells more sensitive to stress.^27^ Moreover, mutations in *TIA*‐*1* also affect the formation of SGs.^28^ We reported that the level of TIA‐1 in both UBQLN2‐overexpressing wild‐type and P497H mutant cells was decreased to different degrees by SA treatment (Figure 3A, B), which results the suppression of SGs assembly (Figure 6). Meanwhile, in mutant SOD1‐transfected cells, the formation of TIA‐1‐positive stress granules was delayed. Mutation in TIA‐1 slows SG disassembly following heat shock in HeLa cells and decreases TDP‐43 mobility in SGs.^29^ Interestingly, the assembly of SGs was also affected in the overexpression of wild‐type UBQLN2, and there was no significant difference in the level of TIA‐1. This indicates that there is another mechanism which independent of TIA‐1, involved in the regulation of SGs with UBQLN2.

The nuclear RNA‐binding protein TDP‐43 is integrally involved in RNA processing. Cytoplasmic mislocalization and accumulation of TDP‐43 are important features of ALS.^30^, ^31^ The subcellular localization of TDP‐43 and related RNA‐binding proteins is critical for neuron function and survival. Disruption of the TDP‐43 nuclear localization signal enhances neurotoxicity. Inducing macroautophagy reduces the concentration of cytoplasmic TDP‐43, which can prevent neurodegeneration in ALS and FTD models and prolong cell survival.^32^ Increasing evidence indicates that nuclear and cytoplasmic transport dysfunction is a conservative approach to age‐dependent susceptibility to neurodegenerative degeneration and neurodegenerative diseases. Decreased expression of nuclear cytoplasmic transport components is a decisive feature of senescent fibroblasts and neurons that differentiate directly from senescent fibroblasts.^33^ This may explain the age‐dependent onset of neurodegeneration in ALS, FTD, and other neurodegenerative diseases. Our results show that overexpression of UBQLN2 can promote the mislocalization of TDP‐43 and SA treatment can also enhance TDP43 cytoplasmic localization. This suggests that the accumulation of UBQLN2‐positive inclusion bodies in UBQLN2‐related ALS patients may cause mislocalization of TDP‐43, thereby inducing neuronal degeneration. Interestingly, a recent study revealed that TDP‐43 colocalized with SGs in the cytoplasm of yeast cells and facilitated their disassembly.^34^

In the present study, neither wild‐type UBQLN2 nor the P497H mutant affected the phosphorylation of eIF2α. This indicates that UBQLN2 negative regulation of SGs could be independent of phospho‐eIF2α. 4E‐BP1 is a downstream molecule of mTOR signaling pathway, and the increased level of phosphorylated 4E‐BP1 is associated with mTOR activation. Inhibition of mTOR could decrease the level of phosphorylated 4E‐BP1 and inhibit the formation of SGs.^35^ The activation of mTOR requires the involvement of UBQLNs. In drosophila, the nonsense mutation of UBQLN1 reduces mTOR activity and the phosphorylation level of 4E‐BP1. In Daoy cells, loss of UBQLNs reduces mTORC1 activity.^12^ In our study, a decrease in phospho‐4E‐BP1 after SA treatment was not observed in wild‐type UBQLN2, surprisingly, we found that P497H UBQLN2 downregulated phospho‐4E‐BP1 after SA treatment (Figure 4C, D), and the number of SGs was also decreased (Figure 2A‐C). It indicates that mTOR may be involved in the inhibition of SGs formation by UBQLN2‐P497H.

Many studies have shown that the broad prospects of mouse models in the mechanism and therapy research of ALS.^36^, ^37^, ^38^, ^39^, ^40^, ^41^ It is reported that UBQLN2‐P497H overexpression mice have pathological phenomena of neuron loss, behavioral defects, and protein aggregation.^42^, ^43^, ^44^ Another UBQLN2‐P497H mice expressing about 20% endogenous level using NF‐H neuron promoter was reported, and no obvious pathological phenomenon was found.^45^ The UBQLN2‐P506T knock in mice had cognitive defects and hippocampal endosomes, but no behavioral abnormalities were found.^46^ These suggest that UBQLN2 mutation may lead to the disease in a harmful and expression level‐dependent manner. Considering the difficulty of stressing in animal models, there are few in vivo studies focused SGs dynamic in ALS mouse model. Our finding that the role of UBQLN2 mutant in regulating SGs was different from wild‐type in vitro needs further verification in animal models.

## 5 CONCLUSIONS

In summary, ubiquilin 2 colocalizes with the SG component proteins G3BP1, TIA‐1, ATXN2, and PABPC1. It participates in regulating SG dynamics. And UBQLN2 mutation affects the assembly of stress granules by regulating TIA‐1. In addition, the overexpression of the UBQLN2 P497H mutant inhibited the phosphorylation of 4E‐BP1 and affected the nuclear and cytoplasmic distribution of TDP43. These provide new insights into the role of UBQLN2 in oxidative stress and the pathogenesis of ALS.

## CONFLICT OF INTEREST

The authors declare no conflict of interest.

## AUTHOR CONTRIBUTIONS

GP, AG, HN, LC, and YC performed the experiments. MZ, JL, and YZ analyzed the data. XL and GP wrote the manuscript with support from DL and ML. XL and ML designed and supervised the study. All authors contributed to the article and approved the submitted version.

## INSTITUTIONAL REVIEW BOARD STATEMENT

Not applicable.

## INFORMED CONSENT STATEMENT

Not applicable.

## Supporting information

Supplementary Material

Supplementary Material

## Data Availability

The authors confirm that the data supporting the findings of this study are available within the article and its supplementary materials.
