# Supplementary material for: Amyotrophic lateral sclerosis (ALS) linked mutation in Ubiquilin 2 affects stress granule assembly via TIA‐1
Source: CNS Neurosci Ther. 2021 Nov 8;28(1):105–15. doi: 10.1111/cns.13757 (PMC8673703; doi:10.1111/cns.13757)
Supplement: Supplementary file 1 — Supplementary Material [file CNS-28-105-s002.docx]

Supplementary Material
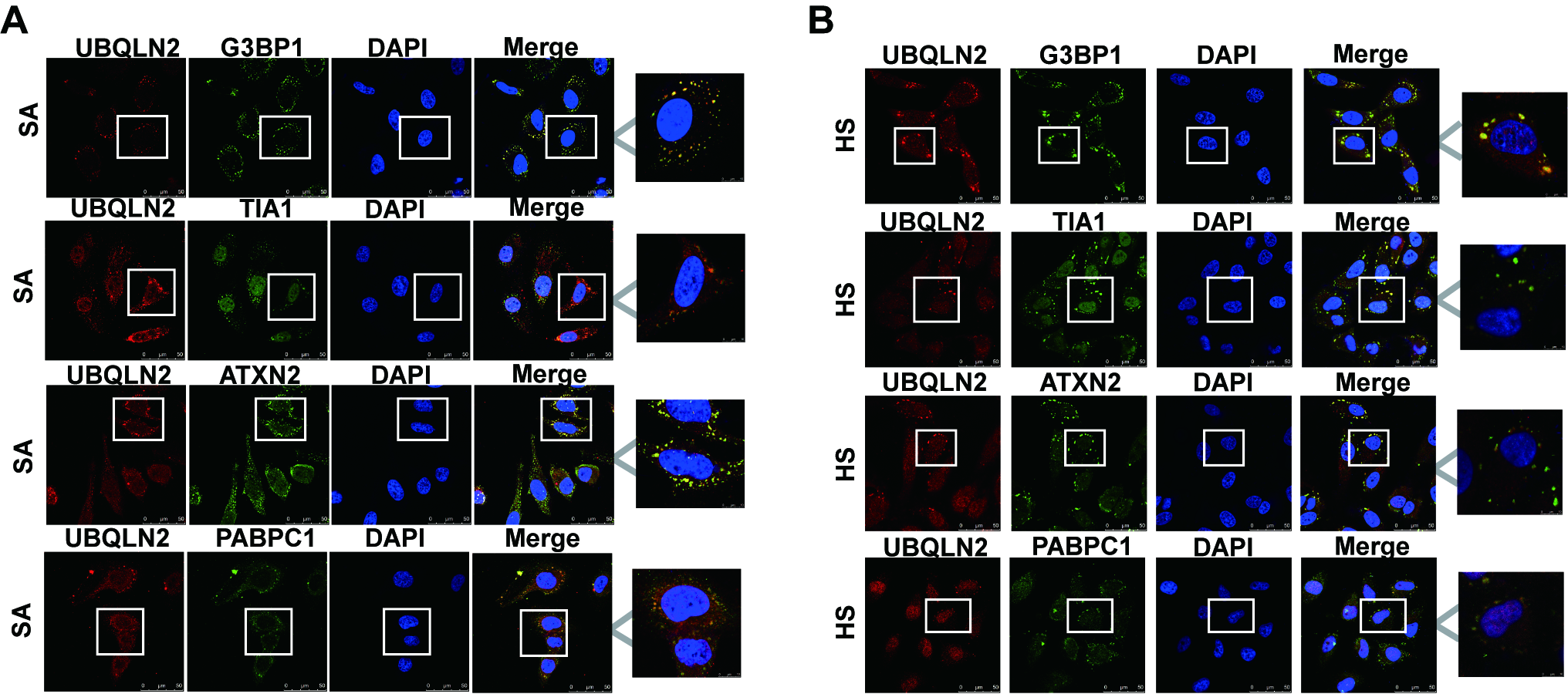


**Figure S1.** Detection of the co-localization of UBQLN2 and SGs component protein in Hela cells. A: Immunofluorescence showed that endogenous UBQLN2 was co-located with SGs components G3BP1, TIA-1, ATXN2, and PABPC1. B: In Hela cells, after 60 min of HS treatment, endogenous UBQLN2 was co-localized with G3BP1, TIA-1, ATXN2, and PABPC. Regions within the boxes are magnified in the figure. Scale 50 μm.

**
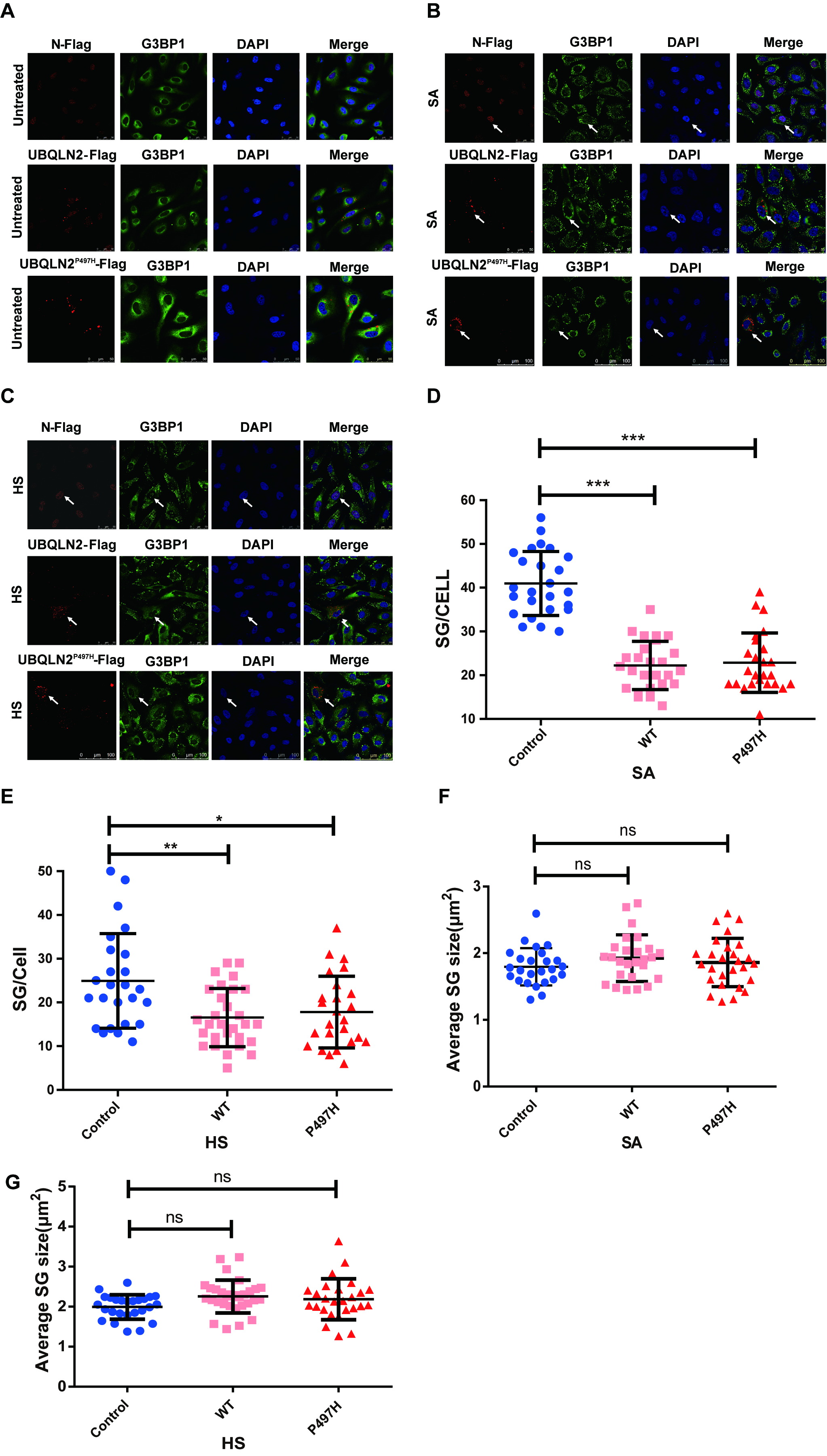
**

**Figure S2.** Regulation of UBQLN2 on SGs in HeLa cells. A-C: After expressing UBQLN2 wild type and P497H mutant, HeLa was not treated (A) or subjected to SA (B) and HS (C) stress treatment for immunofluorescence, and G3BP was used as the marker protein of SGs. Arrows indicate the status of G3BP in the transfected cells. Scale 50 μm. D-E: after SA or HS treatment, 25 transfected cells were randomly selected and the number of SGs particles in each cell was counted. F-G: 10 transfected cells were randomly selected and the area of each SGs particle was counted. One-way ANOVA was used to test the significance of Tukey. ***P < 0.001, **P <0.01, *P < 0.05. ns, not significant.


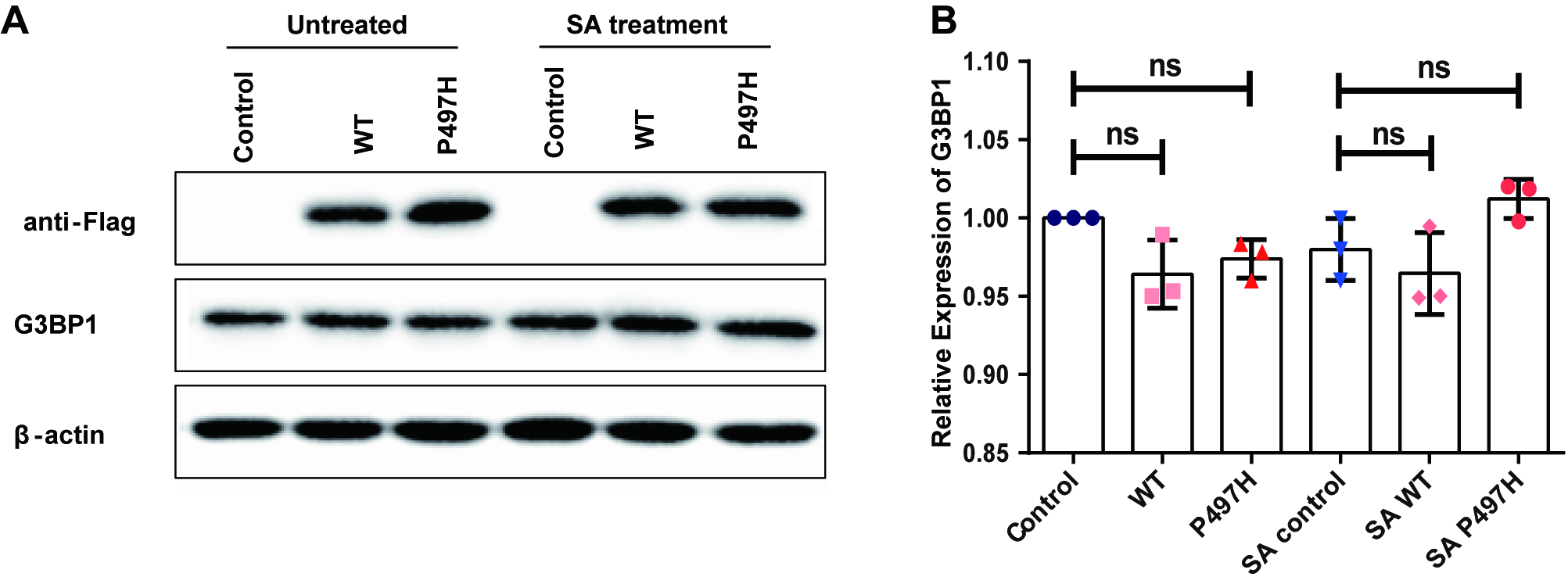


**Figure S3:** Detection of SG component proteins. A-B: After SA treatment for 30 min, the expression level of G3BP1 in the control (N-flag), WT (UBQLN2-flag) and P497H (UBQLN2-P497H-flag) mutant groups was detected by immunoblotting (B) and statistical analysis was performed (B). One-way ANOVA was used to test the significance of Tukey’s test results. ns, not significant.


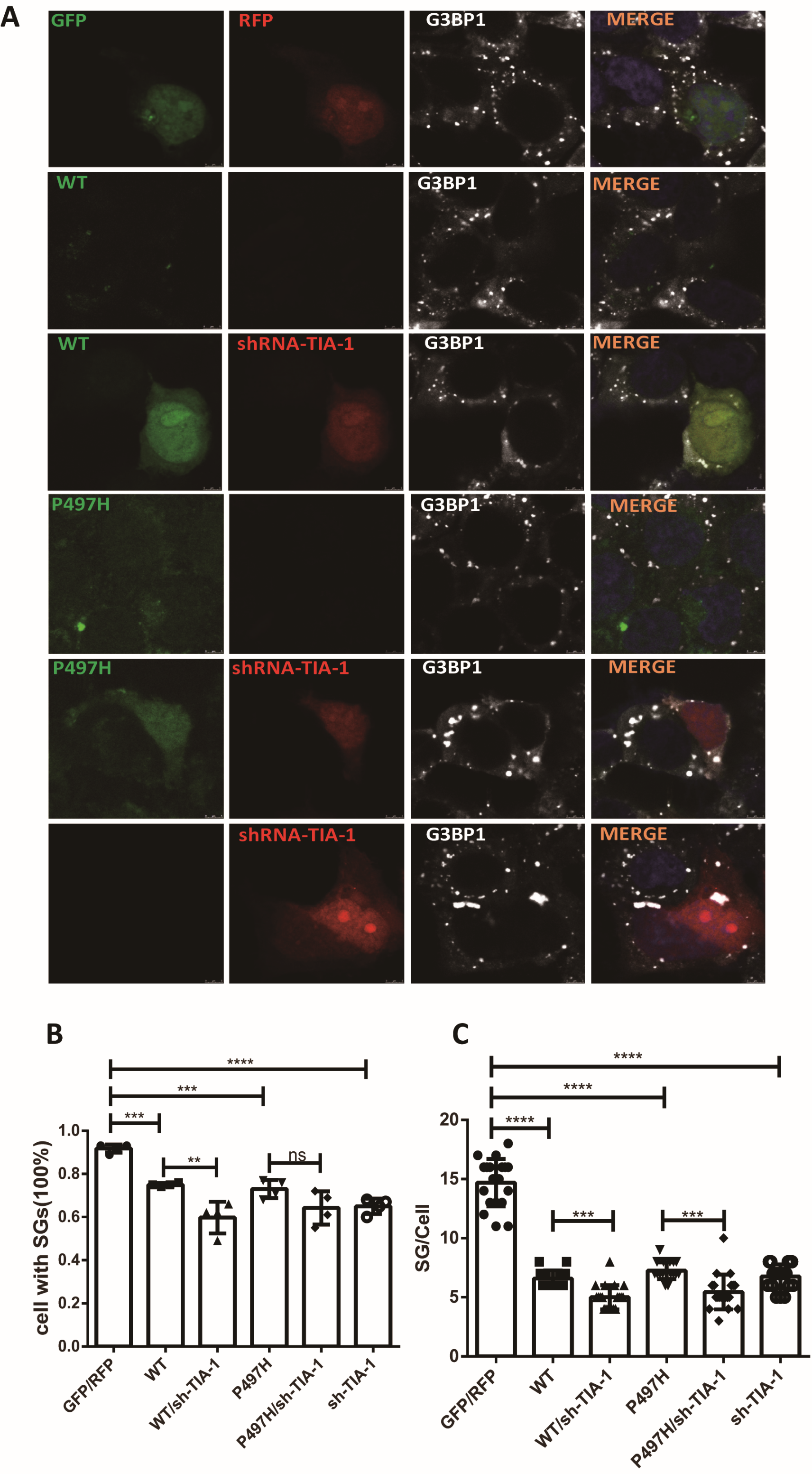


**Figure S4.** Examination of the effect of UBQLN2 on stress granule when knockdown TIA1. A: 293T cells were transfected with GFP, RFP, WT, P497H, or shRNA-TIA1 plasmids. Meanwhile, WT and shRNA-TIA1, P497H and shRNA-TIA1 plasmids were co-transfected into 293T cells for immunofluorescent. Cells treated with SA for 30 min. Scale, 5 μm. B: Percentage of cells containing stress granules in cells treated with control or experimental groups after 30 min SA treatment. C: Significant differences in the number of SGs per cell were observed after 30 min of SA treatment. One-way ANOVA was used to test the significance of Tukey’s test results. ****P < 0.0001; ***P < 0.001; **P <0.01; ns, not significant.


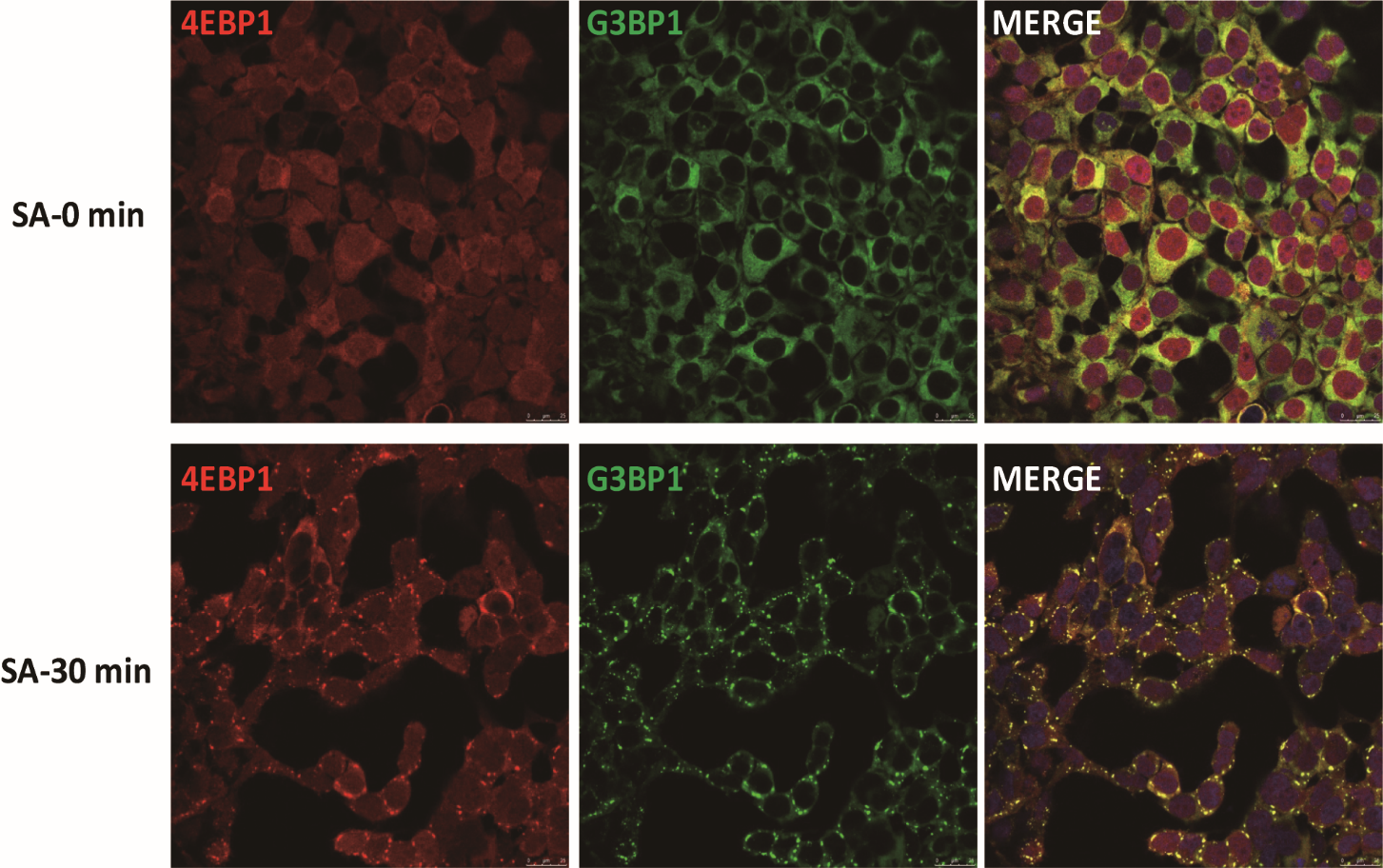


**Figure S5.** Detection of the localisations of 4E-BP1 and SGs. Confocal microscopic images of HEK293T cells without treatment or 30 min of arsenite treatment. The endogenous 4E-BP1 (red) and G3BP1 (green) were stained, and images were acquired with LAS X SP-5 confocal microscope. The nuclei were stained with DAPI. Scale bars 25 μm.


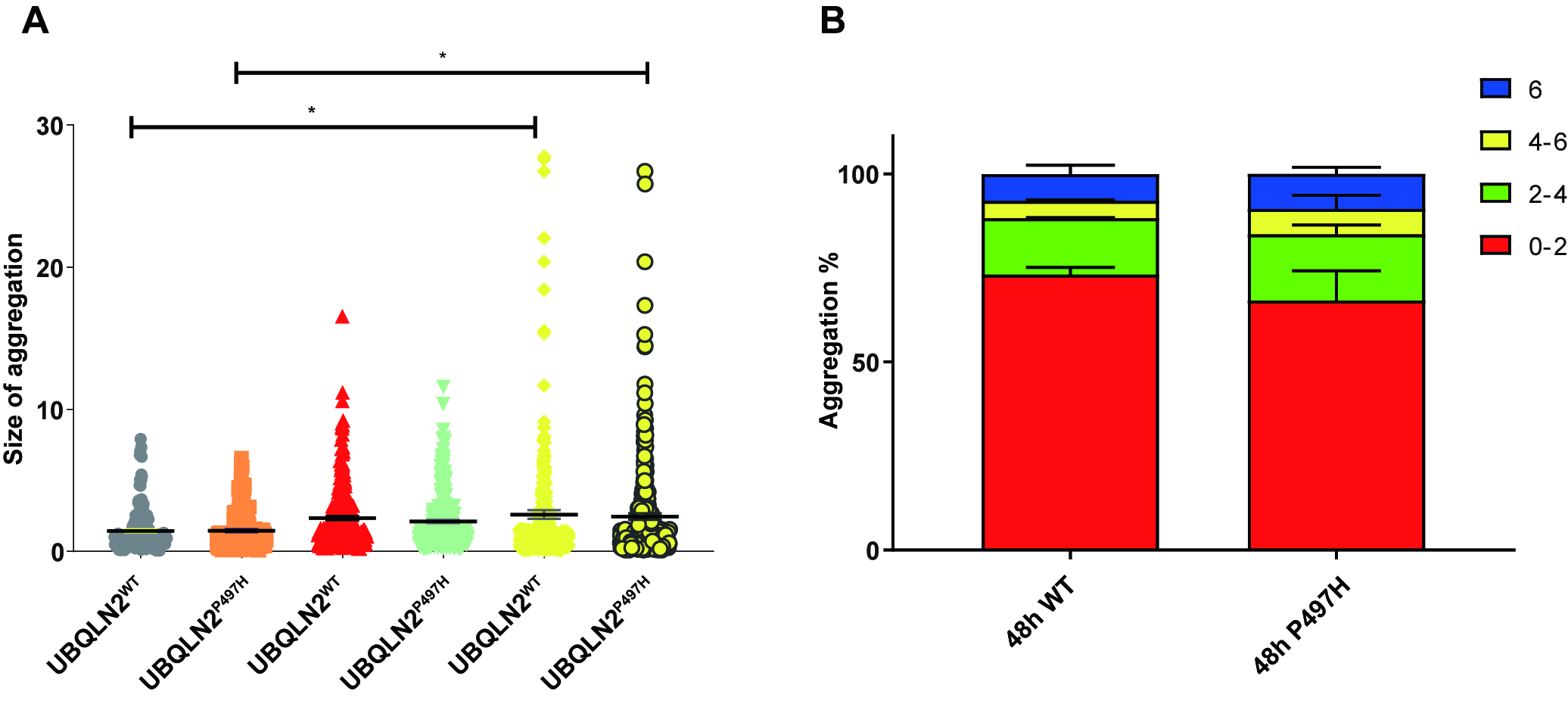


**Figure S6.** Detection of the aggregation size of UBQLN2 wild-type and P497H mutant. A: HEK293T cells were transitively transfected with wild-type (UBQLN2-GFP) and P497H mutant (UBQLN2P497H-GFP) plasmids, and UBQLN2 aggregation was formed at 24h, 36h and 48h. The aggregation area was counted by image J, and transfected cells were randomly selected for statistical analysis of at least 100 aggregation areas. One-way ANOVA was used to test the significance of Tukey. *P < 0.05. B: The areas gathered at 48h were classified into ranges 0-2, 2-4, 4-6 and greater than 6 μm^2^, and the percentages of each range were calculated.
