# Supplementary material for: Amyotrophic lateral sclerosis (ALS) linked mutation in Ubiquilin 2 affects stress granule assembly via TIA‐1
Source: CNS Neurosci Ther. 2021 Nov 8;28(1):105–15. doi: 10.1111/cns.13757 (PMC8673703; doi:10.1111/cns.13757)

Figure 3A

Anti-Flag（UBQLN2-flag）： 67KD

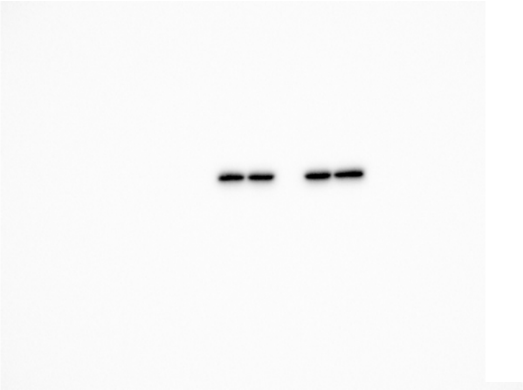

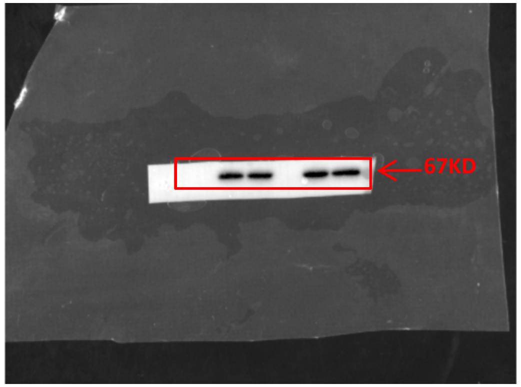

TIA-1： 43KD

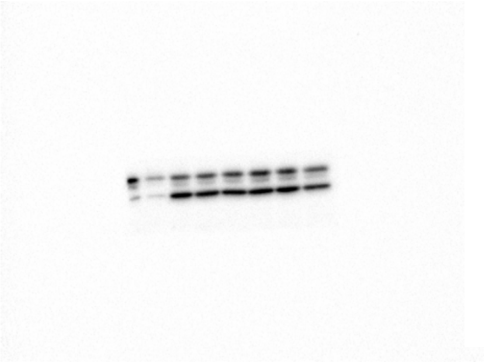

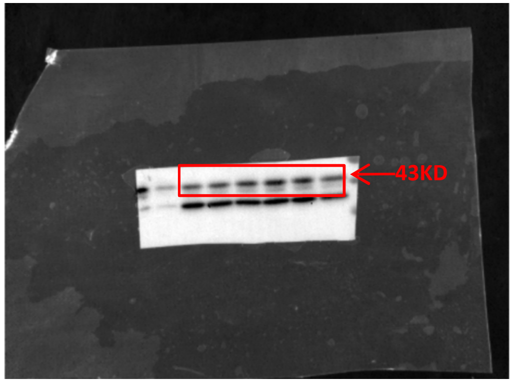

β-actin: 42kD

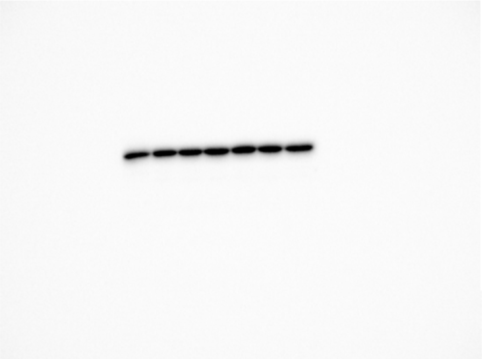

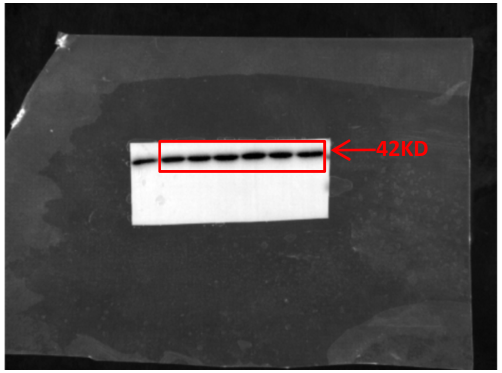

Figure 3C

UBQLN2：65KD

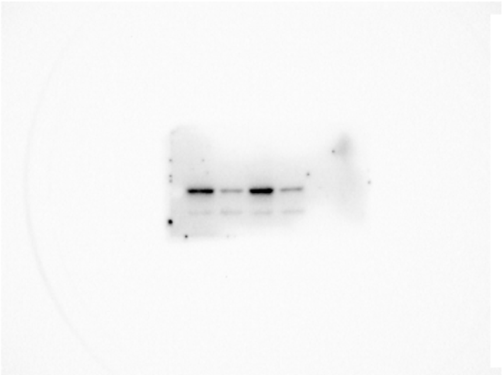

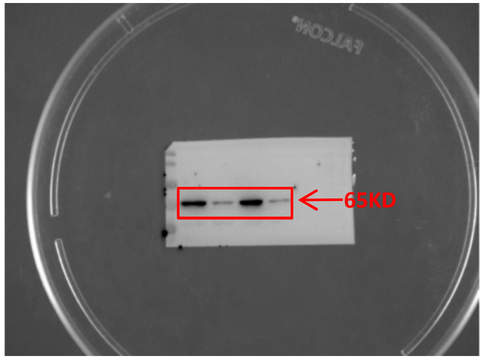

TIA-1：43KD

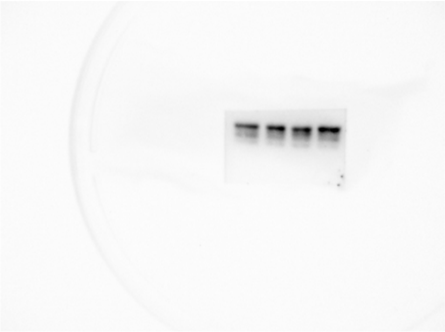

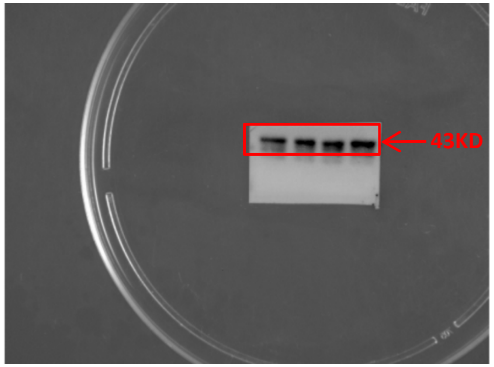

α-tubulin: 55kD

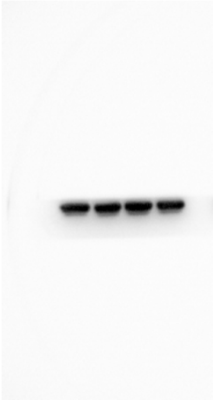

Figure 4A

Anti-Flag（UBQLN2-flag）： 67KD

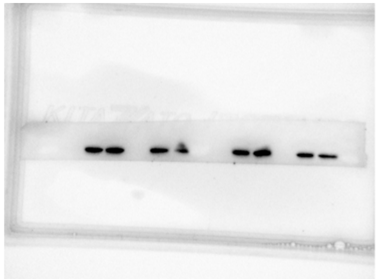

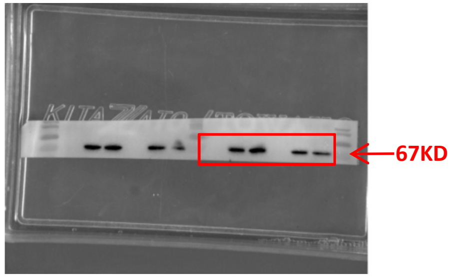

p-eIF2α： 38KD

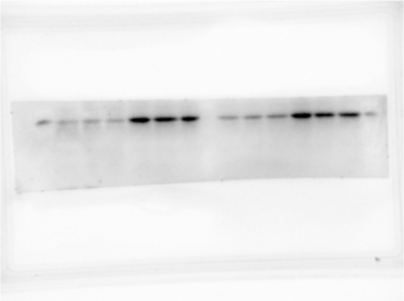

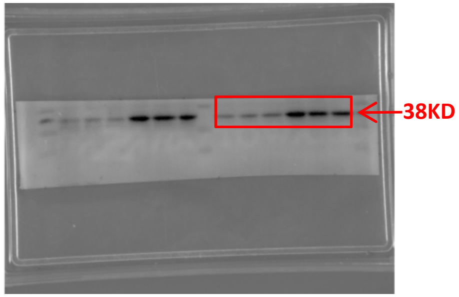

eIF2α: 38kD

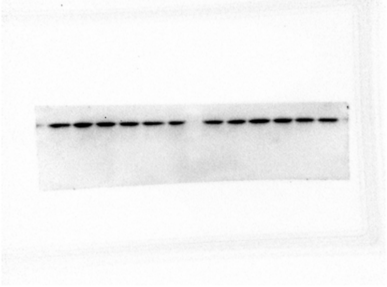

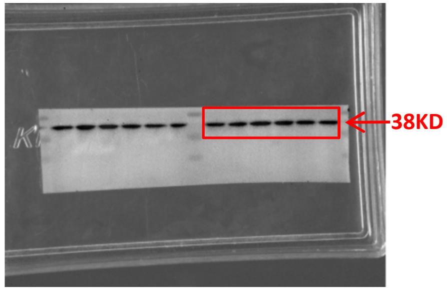

tubulin: 55kD

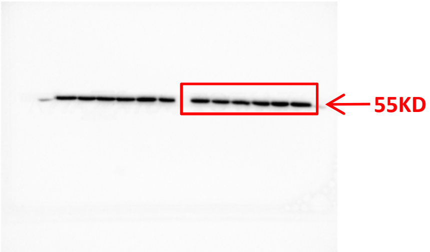

Figure 4C

Anti-Flag（UBQLN2-flag）： 67KD

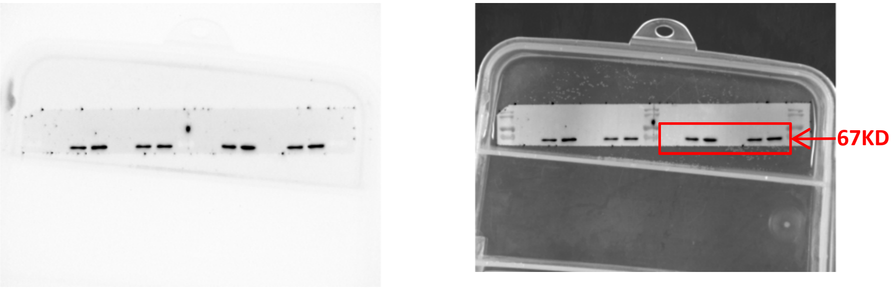

p-4EBP1（Ser65）： 18KD

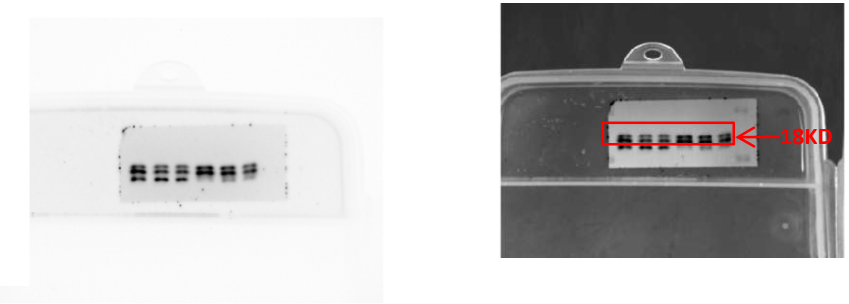

4EBP1: 18kD

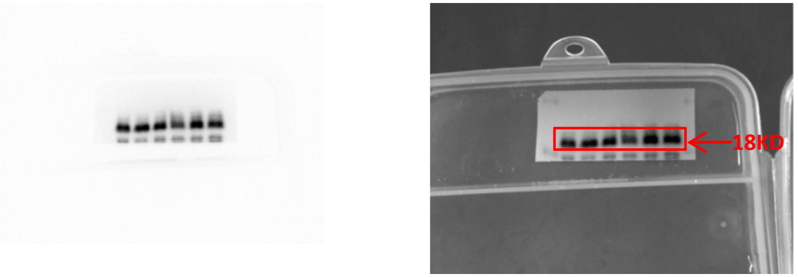

tubulin: 55kD

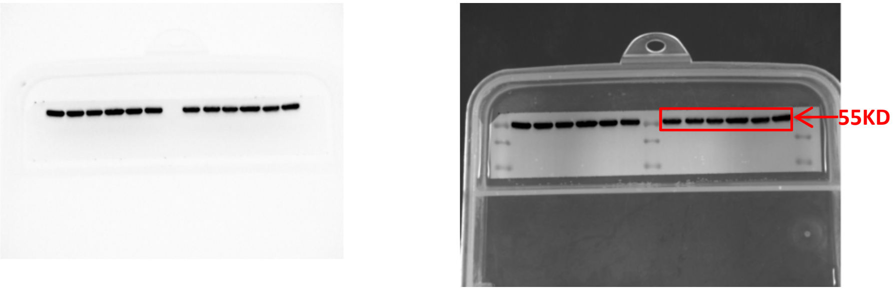

Figure 5A

Anti-Flag（UBQLN2-flag）： 67KD

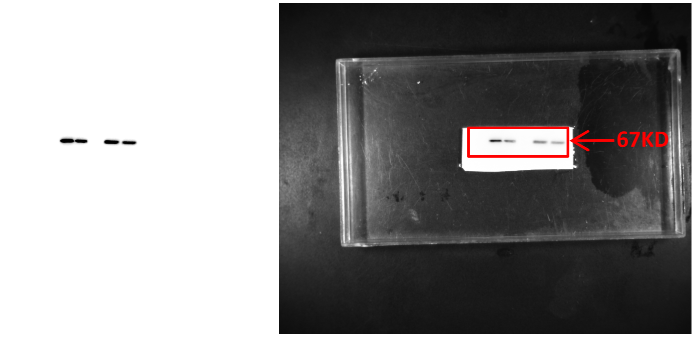

β-actin: 43kD

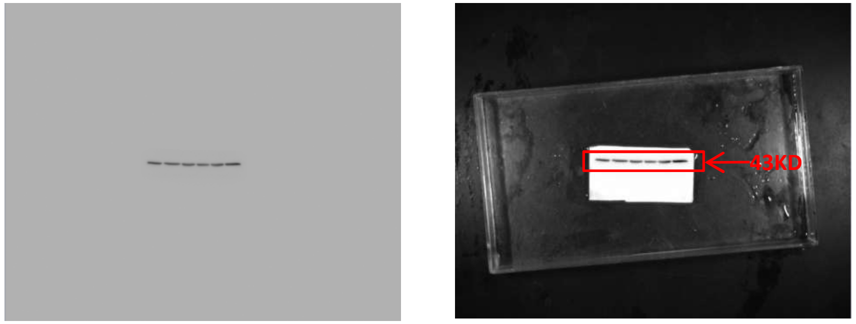

FUS: 70kD

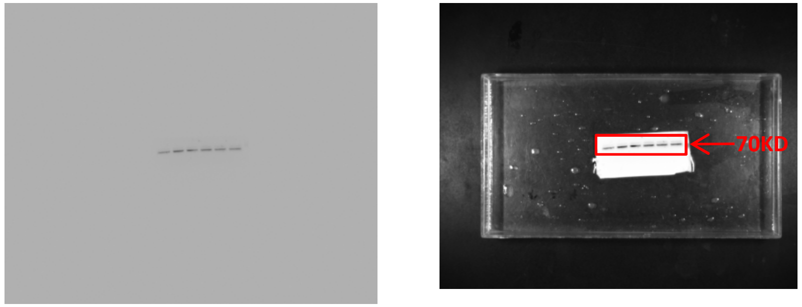

C9orf72：50KD

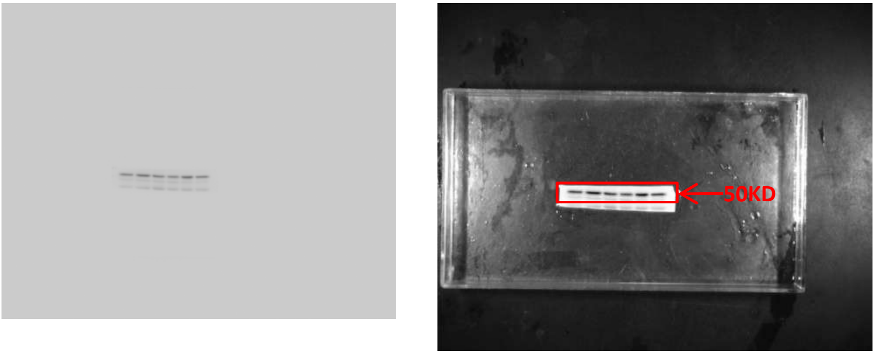

Figure 5C

Anti-Flag（UBQLN2-flag）： 67KD

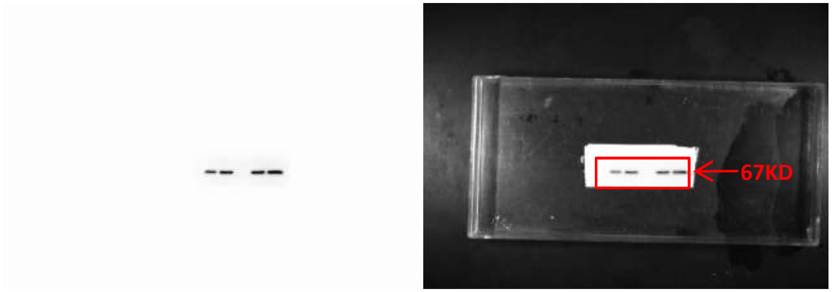

β-actin: 43kD

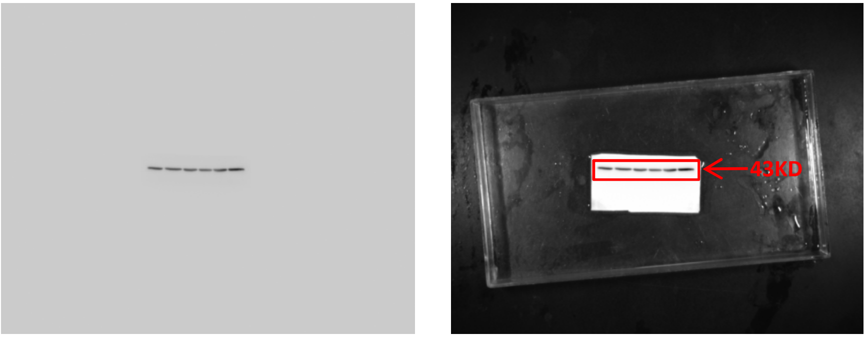

Figure 5E

Anti-Flag（UBQLN2-flag）： 67KD

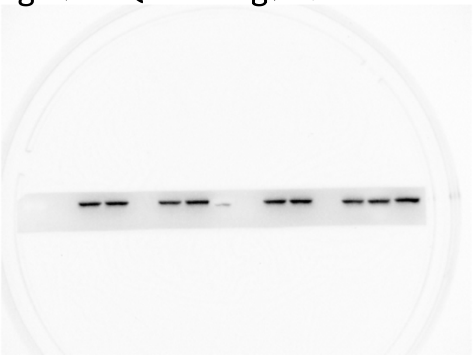

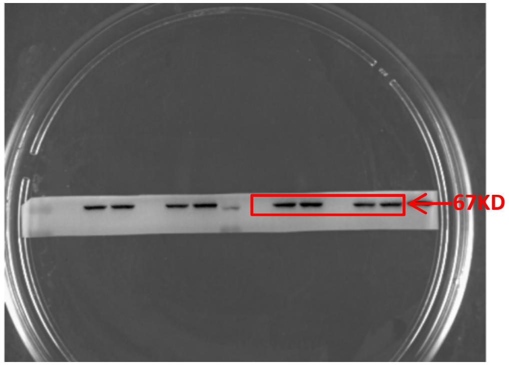

TDP-43： 43KD

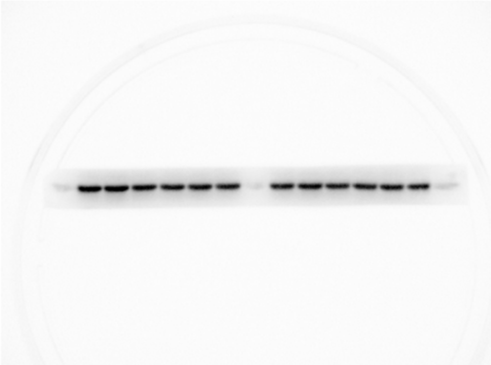

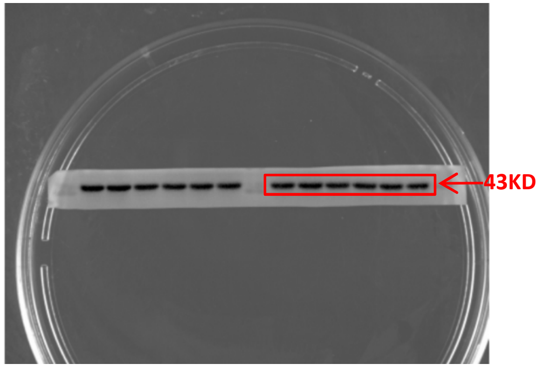

GAPDH： 37KD

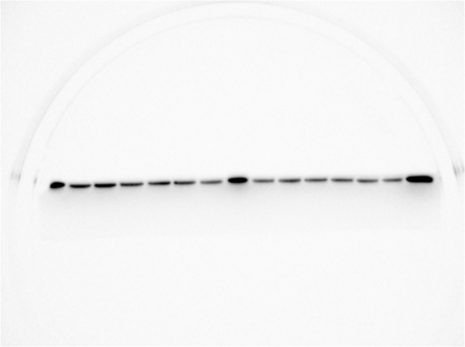

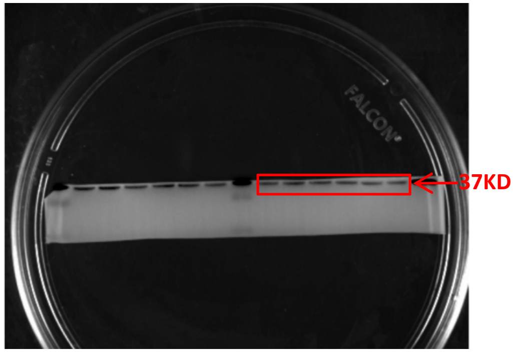

Figure 5G

TDP-43：43KD

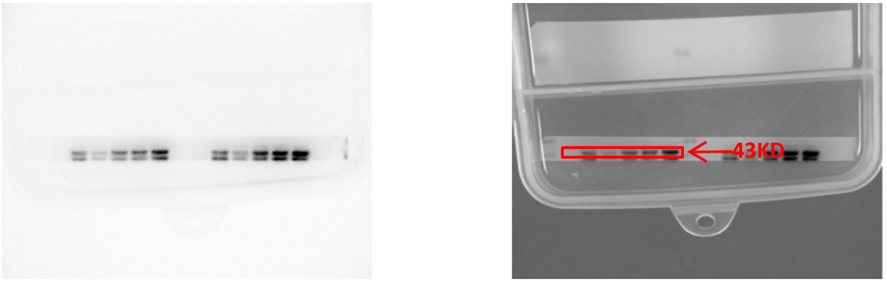

TDP-43：43KD

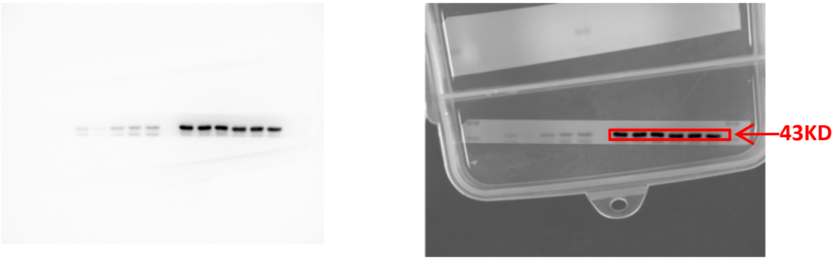

GAPDH: 37kD

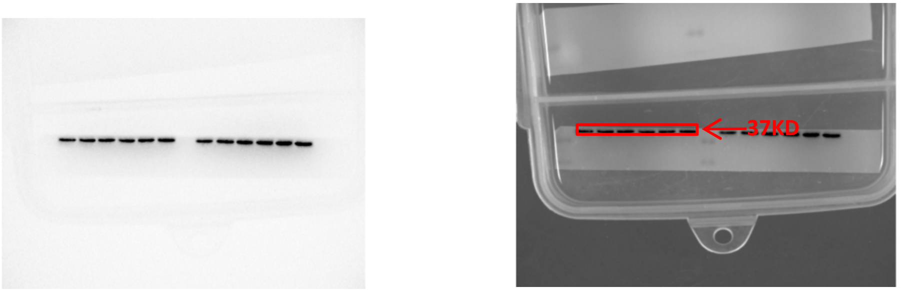

H3：17KD

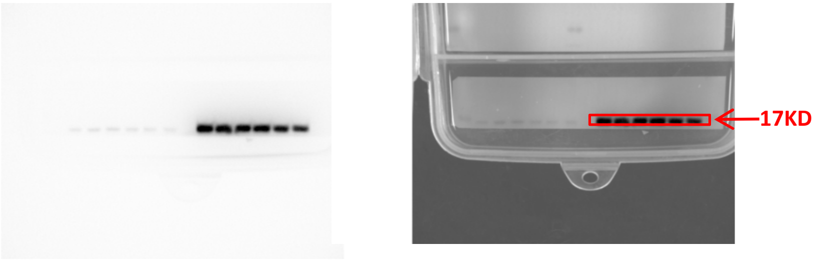

Figure S3A

Anti-Flag（UBQLN2-flag）： 67KD

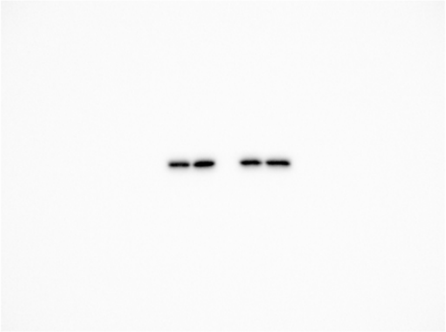

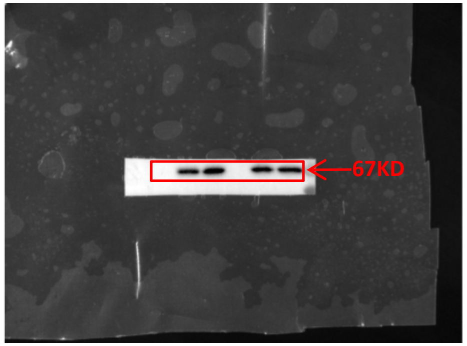

G3BP1： 68KD

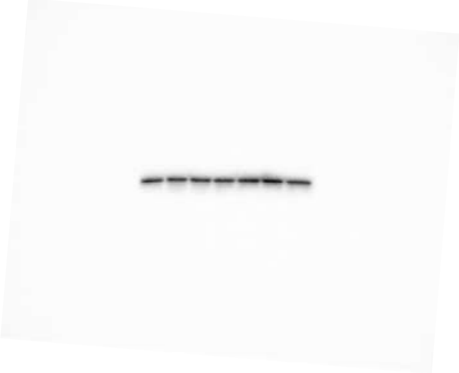

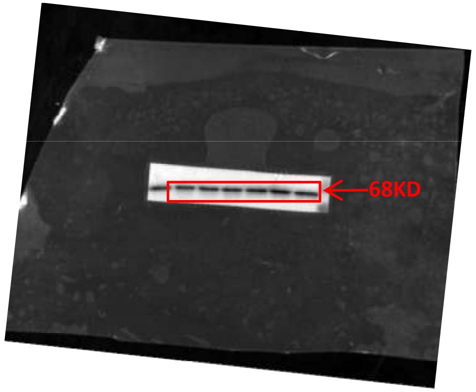

β-actin： 43KD

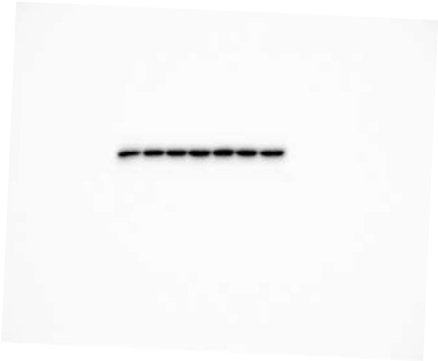

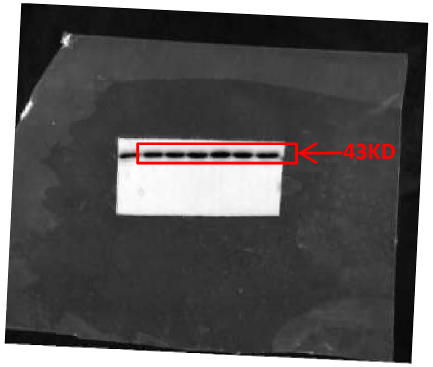

Supplement: Supplementary file 2 — Supplementary Material [file CNS-28-105-s001.pdf]
